# Impact of post-COVID-19 lung damage on pulmonary function, exercise tolerance and quality of life in Indian subjects

**DOI:** 10.1371/journal.pgph.0002884

**Published:** 2024-02-01

**Authors:** Devasahayam Jesudas Christopher, Barney T. J. Isaac, Flavita Benna John, Deepa Shankar, Prasanna Samuel, Richa Gupta, Balamugesh Thangakunam

**Affiliations:** 1 Department of Pulmonary Medicine, Christian Medical College, Vellore, Tamil Nadu, India; 2 Department of Pulmonary Medicine, Respiratory Therapist & Research Co-ordinator, Christian Medical College, Vellore, Tamil Nadu, India; 3 Department of G.I. Sciences & Department of Biostatistics, Christian Medical College, Vellore, Tamil Nadu, India; 4 Professor and Head of Respiratory Medicine, Christian Medical College, Vellore, Tamil Nadu, India; PLOS: Public Library of Science, UNITED STATES

## Abstract

After recovery from COVID-19, there is data to suggest potential long-term pulmonary sequelae and associated impairment of functional capacity. This cross-sectional study was designed to assess the impact on respiratory function in a cohort of Indian subjects. Subjects who had recovered from COVID-19 were recruited. Clinical symptoms, pulmonary function test results, 6-minute walk test (6MWT) results, St George’s Respiratory questionnaire (SGRQ) and chest radiographs were obtained. Information on the COVID-19 illness during hospitalization, baseline laboratory biomarkers and the disease severity categories as outlined by WHO (asymptomatic, mild, moderate, severe and critical), were retrieved from the hospital records. The ‘COVID pneumonia’(WHO category moderate, severe & critical) group was compared with the ‘Mild COVID’ (WHO category mild) group and likewise, the WHO category moderate and the WHO category severe/critical groups were compared. In 207 subjects, whose mean age was 48.7 years were assessed after an average of 63 days from onset of symptom, 35% had TLC< 80% (restrictive defect), 8.3% had FEV1/FVC<70% (obstructive defect) and 44.4% had diminished DLCO<80% (diffusing capacity). The ‘COVID-19 pneumonia’ group when compared to the ‘mild COVID-19’ group, had lower FVC% (77.85 VS 88.18; P = 0.001), TLC% (79.48 VS 87.91; P = 0.0002), DLCO% (75.30 VS 89.20; P<0.0001) and DLCO/VA% (105.6 VS 111.8; P = 0.032), decreased minimum oxygen saturation (94.89 VS 97.73; P<0.0001) and more subjects had a drop in saturation of ≥ 4% (21.69% VS 4.84%; P = 0.001) during the 6MWT, and a greater mean total SGRQ score (29.2 VS 11.0; P<0.0001). To our knowledge, this is the first such report on Indian subjects. We have shown that post-COVID-19 lung damage leads to significant impairment of lung function, quality of life and effort tolerance.

## Introduction

COVID-19 is caused by severe acute respiratory syndrome coronavirus 2 (SARS-CoV-2), which is a novel coronavirus. The commencement of the global pandemic was in Wuhan, China, in December 2019, and it has spread worldwide [1]. As of 1st June 2023, the total case tally in India stands at 44,990,876, including 3,925 active cases, 44,455,079 discharged and 531,872 deaths, according to the Ministry of Health and Family Welfare [2]. Thankfully, the cases of COVID-19 infections have receded globally.

SARS-CoV-1 and MERS-CoV, which are genetically comparable to SARS-CoV-2, were reported to cause severe lung disease. Previous studies have reported reticular changes two weeks after symptom onset in SARS-CoV-1 that didn’t resolve in half of the patients after a month [3]. Long-term follow-up, 15 years after they had been infected with SARS-CoV-1, there were interstitial abnormalities in 4.6% of patients [4].

In people recovering from COVID-19, there is evidence of potential long-term pulmonary sequelae and associated lung function impairment [5]. The most severe illness in the context of SARS-CoV-2 infection is acute respiratory distress syndrome (ARDS). In some, the ARDS may result in fibrotic interstitial lung disease.

In a cohort of 159 autopsies, of patients with ARDS, Thille and coworkers reported that alveolar damage either resolved or progressed to fibrosis [6]. In their study, fibrosis developed in 4% of patients with less than one week of the disease, 24% of patients with 1–3 weeks and 61% with more than three weeks [6].

This study was designed to study the impact of post-COVID-19 lung damage in patients who have recovered from COVID-19 infection, as assessed by; lung function testing, exercise tolerance, chest radiography and quality of life measurement.

## Material and methods

### Study subjects

Patients managed for their COVID illness at our hospital who provided consent to participate in the trial, conducted by the Department of Pulmonary Medicine, between 11^th^ August 2020 to 14^th^ January 2021.

### Study design

This was a cross-sectional observational study. The sample size was calculated based on a previous study which showed low total lung capacity (TLC) in 25% of patients after COVID-19 infection [7]. We required TLC assessment in 153 patients, to achieve the outcome of the proportion of patients with restrictive ventilatory defect with a precision of 7%.

### Ethics statement

This study was conducted as per the amended Declaration of Helsinki. Approval for the study was obtained by the institutional review board of the Christian Medical College, Vellore (IRB No- 12975). The subjects were enrolled in the study after obtaining formal written consent.

### Methods

Consenting adult patients (≥18 years of age) diagnosed with COVID-19 (positive SARS-CoV-2 RNA detection by throat swab), who had recovered from the illness, were approached for participation in the trial. The recruitment occurred between 11^th^ August 2020 to 14^th^ January 2021, and covered patients who were affected in the first wave of the COVID-19 pandemic in India. After obtaining informed consent, a clinical research form was administered to collect relevant clinical information and thereafter the subjects underwent pulmonary function testing, exercise tolerance was measured by 6-minute walk test (6MWT), chest radiography and assessment of health-related quality of life score was assessed using St George’s Respiratory questionnaire (SGRQ).

The case report form captured demographic information, current symptoms and comorbidities including pre-existing lung diseases. Information pertaining to hospitalization for the acute illness: symptoms, need for oxygen supplementation, non-invasive or invasive ventilation, the WHO disease severity category (asymptomatic, mild, moderate, severe and critical) [8], and laboratory biomarkers were retrieved from the hospital electronic medical records. For the purpose of comparison, they were regrouped into ‘mild COVID-19’ (WHO category mild) and ‘COVID-19 pneumonia’ (WHO category moderate, severe & critical). The WHO category moderate was also compared with the WHO category severe/critical.

#### Pulmonary function tests

Pulmonary function tests (PFT) were performed using Master Screen Body (Jaeger, Wurzburg, Germany), by experienced respiratory therapists. The following parameters of the PFT were measured: FVC (Forced Vital Capacity), FEV1 (Forced Expiratory Volume at the end of the first second), FEV1/FVC (ratio of Forced Expiratory Volume at the end of the first second to the Forced Vital Capacity), TLC (total lung capacity), RV (Residual Volume), DLCO (Diffusing capacity of lung for carbon monoxide), DLCO/VA (Diffusing capacity adjusted for the alveolar volume ventilated). All PFT measurements were expressed as actual measured values, as well as percentages predicted of normal values.

#### Six-minute walking test

6-MWT was performed in a 30 meter corridor by respiratory therapists according to American Thoracic Society guidelines [9]. The distance walked in meters, the lowest recorded saturation, the distance-saturation product (DSP); which is the product of distance walked in meters with the lowest recorded saturation, 6-minute walk work (WW); which is the product of the distance walked and the weight of the patient in Kgs, and the walk distance as percentage of predicted, were calculated.

#### Chest radiographs

Chest radiographs were scored using Radiographic Assessment of Lung Edema (RALE) score proposed by Warren et al. in 2018 [10].

#### St. George’s respiratory questionnaire (SGRQ)

SGRQ is a disease-specific instrument created to assess the effects of obstructive airway disease on a patient’s overall health, daily life, and perceived well-being. They are assessed across three domains: symptoms, activity and impact. We used the total scores to divide the patients into more symptomatic (SGRQ ≥25) and less symptomatic (SGRQ < 25) as per GOLD [11].

#### Statistical analysis

Analysis was done using Stata (Version 11; StataCorp, College Station, Texas, US). The distribution of continuous variables were assessed visually using histograms with normality curves. Those that were normally distributed were expressed as mean and standard deviation. For skewed continuous variables, median and interquartile range (IQR) (difference between 75th and 25th percentiles, or between upper and lower quartiles, IQR = Q₃ − Q₁) was used. Categorical variables were expressed as frequencies and percentages.

In the univariate analysis, for normally distributed continuous variables independent t-test, which is a parametric test, was used. For skewed continuous variables Mann-Whitney U test, which is a non-parametric test, was used. For categorical variables, Pearson’s chi-square test was used except in situations when the occurrences were less than 5, when Fisher’s exact test was used. Two-sided P value < 0.05 was taken as statistically significant.

The outcome or dependent variables were COVID-19 pneumonia, WHO category—mild, moderate and severe/critical. The independent variables were demographics (age, gender), BMI, risk factors (smoking history and comorbidities), clinical characteristics (cough and shortness of breath) and biomarkers (Neutrophil/lymphocyte ratio, C reactive protein (CRP), D dimer and Lactate Dehydrogenase (LDH)], all pulmonary function test variables (FVC, FEV1, FEV1/FVC, TLC, RV/TLC, DLCO, DLCO/VA), 6-MWT variables (walk distance, distance saturation product, walk work, oxygen saturation), SGRQ variables (total score and domain scores-symptom, activity, impact) and chest x-ray RALE score. The comparisons were made to test if the independent variables were significantly different between the specified groups and to assess if any of them were associated with a more severe COVID-19 illness.

## Results

We recruited 207 subjects with a mean age of 48.7 years and of whom 141 (68.1%) were males. The subjects were distributed to the WHO disease severity categories as follows: mild 124 (59.9%), moderate 41 (19.8%) and severe & critical 42 (20.3%) categories. Thus, the comparison groups ‘mild COVID-19’ (WHO category mild) had 124 (59.9%) subjects and ‘COVID-19 pneumonia group’ (WHO category moderate, severe and critical) had 83 (40.1%) subjects. The mean time from the onset of symptoms to the study assessments was 63 days. Table 1 compares the demographic data, comorbidities, respiratory symptoms (dyspnoea & cough) and blood biomarkers during the acute illness, across the disease severity categories. The mean age of the patients in the ‘COVID-19 pneumonia group’ was higher than the mean age of the ‘mild COVID-19’ group (53.7 VS 45.3; P <0.0001) and the former group had more subjects with BMI >25 than the latter group (79.0% VS 67.5%; P = 0.04).

**Table 1:** Demographics, risk factors and clinical characteristics of subjects across disease severity.

Characteristics N = 207	Total	‘Mild COVID-19’ N = 124	‘COVID-19 Pneumonia’ N = 83	P value	WHO Moderate N = 41	WHO Severe/ Critical N = 42	P value
Age, years (SD), n = 207	48.7 (14.2)	45.3 (14.8)	53.7 (11.8)	<0.0001	55.5 (11.5)	51.9 (11.9)	0.16
Male gender, n (%)], n = 207	141 (68.1)	80 (64.5)	61 (73.5)	0.174	28 (68.3)	33 (78.6)	0.289
BMI, kg/m2*(SD), n = 201	27.7 (4.8)	27.2 (4.5)	28.5 (5.0)	0.058	28.7 (4.8)	28.3 (5.3)	0.75
BMI > 25, n (%)	145 (72.1)	81 (67.5)	64 (79.0)	0.04	33 (84.6)	31 (73.8)	0.314
History of smoking, n (%) n = 198							
Never smoked	178 (89.9)	105 (89.0)	73 (91.3)		35 (92.1)	38 (90.5)	
Ever smoked	20 (10.1)	13 (11.0)	7 (8.8)	0.641	3 (7.9)	4 (9.5)	1
**COMORBIDITIES,** n (%), N = 207							
At least one co-morbidity	150 (72.5)	85 (68.6)	65 (78.3)	0.123	33 (80.5)	32 (76.2)	0.635
Two or more co-morbidities	83 (40.1)	44 (35.5)	39 (47.0)	0.098	20 (48.8)	19 (45.2)	0.746
Diabetes	78 (37.7)	37 (29.8)	41 (49.4)	0.004	18 (43.9)	23 (54.8)	0.322
Hypertension	69 (33.3)	39 (31.4)	31 (37.4)	0.483	16 (39.0)	15 (33.3)	0.589
Chronic Respiratory Disease	60 (29.0)	36 (29.0)	24 (28.9)	0.986	10 (24.4)	14 (33.3)	0.369
Ischemic Heart Disease	17 (8.2)	7 (5.6)	10 (12.1)	0.1	5 (12.2)	5 (11.9)	0.968
Malignancy	9 (4.3)	4 (3.2)	5 (6.0)	0.489	4 (9.8)	1 (2.4)	0.202
Chronic Kidney Disease	4 (1.9)	2 (1.6)	2 (2.4)	1	0 (0.0)	2 (4.8)	0.494
Others	44 (21.3)	25 (20.2)	19 (22.9)	0.638	11 (26.8)	8 (19.1)	0.399
**SYMPTOMATOLOGY, **n (%), N = 207							
Cough	56 (27.1)	23 (18.6)	33 (39.8)	0.001	14 (34.1)	19 (45.2)	0.302
Shortness of breath	102 (49.3)	51 (41.1)	51 (61.5)	0.003	18 (43.9)	33 (78.6)	0.001
**BIOMARKERS,** [median (IQR)]							
Neutrophil/lymphocyte ratio, n = 166	2.7(1.6–5.1)	1.9 (1.3–3.3)	4.4 (2.6–7.1)	<0.0001	3.8 (2.2–5.3)	5.1 (3.1–10.8)	0.0375
CRP, mg/dL, n = 96	6.8 (3.2–33)	3.9 (3.1–10.9)	21.6 (5.4–63)	<0.0001	18.3 (4.2–45.3)	36.9 (17.3–78.0)	0.109
D dimer, ng/ml, n = 161	347 (233–573)	301 (218–496)	452.5 (256–853)	0.0005	349 (243–671)	525 (266–1335)	0.0652
LDH, U/L, n = 104	586.5 (445.5–717)	481 (381–612)	676 (552–819)	<0.0001	602 (517–763)	698 (623–846)	0.0992

BMI (Body Mass Index).

CRP (C- Reactive Protein).

LDH (Lactate Dehydrogenase).

Overall, 72.5% subjects had underlying co-morbidities and of which 40.1% had two or more comorbidities. Common comorbidities included diabetes mellitus (37.7%), systemic hypertension (33.3%), chronic respiratory diseases (29.0%), ischemic heart disease (8.2%), malignancy (4.3%) and chronic kidney disease (1.9%). The ‘COVID-19 Pneumonia’ group had more Diabetes Mellitus (49.4% VS 29.8%; P = 0.004) as compared to the ‘mild COVID-19’ group. There was no difference in the comorbidities between the WHO category moderate and the WHO category severe/critical groups. Only 20(10.1%) subjects were ever-smokers.

There was 49.3% subjects with shortness of breath and 27.1% had cough. More subjects in the ‘COVID-19 pneumonia’ group had shortness of breath (61.5% VS 41.1%; P = 0.003) and cough (39.8% VS 18.6%; P = 0.001). More subjects had dyspnoea in the WHO category severe/critical group compared to the WHO category moderate group (78.6% VS 43.9%; P = 0.001).

The median baseline blood biomarkers; were significantly higher in the ‘COVID-19 pneumonia’ group compared to the ‘mild COVID-19’ group: Neutrophil/Lymphocyte ratio (4.4 VS 1.9; P <0.0001), CRP (21.6 VS 3.9; P <0.0001), D dimer (452.5 VS 301; P = 0.0005) and LDH (676 VS 481; P <0.0001). The WHO category severe/critical had higher Neutrophil/Lymphocyte ratio (5.1 VS 3.8; P = 0.0375) than the WHO category moderate group, while CRP, D dimer and LDH were similar.

### Pulmonary function tests

**Fig 1:**
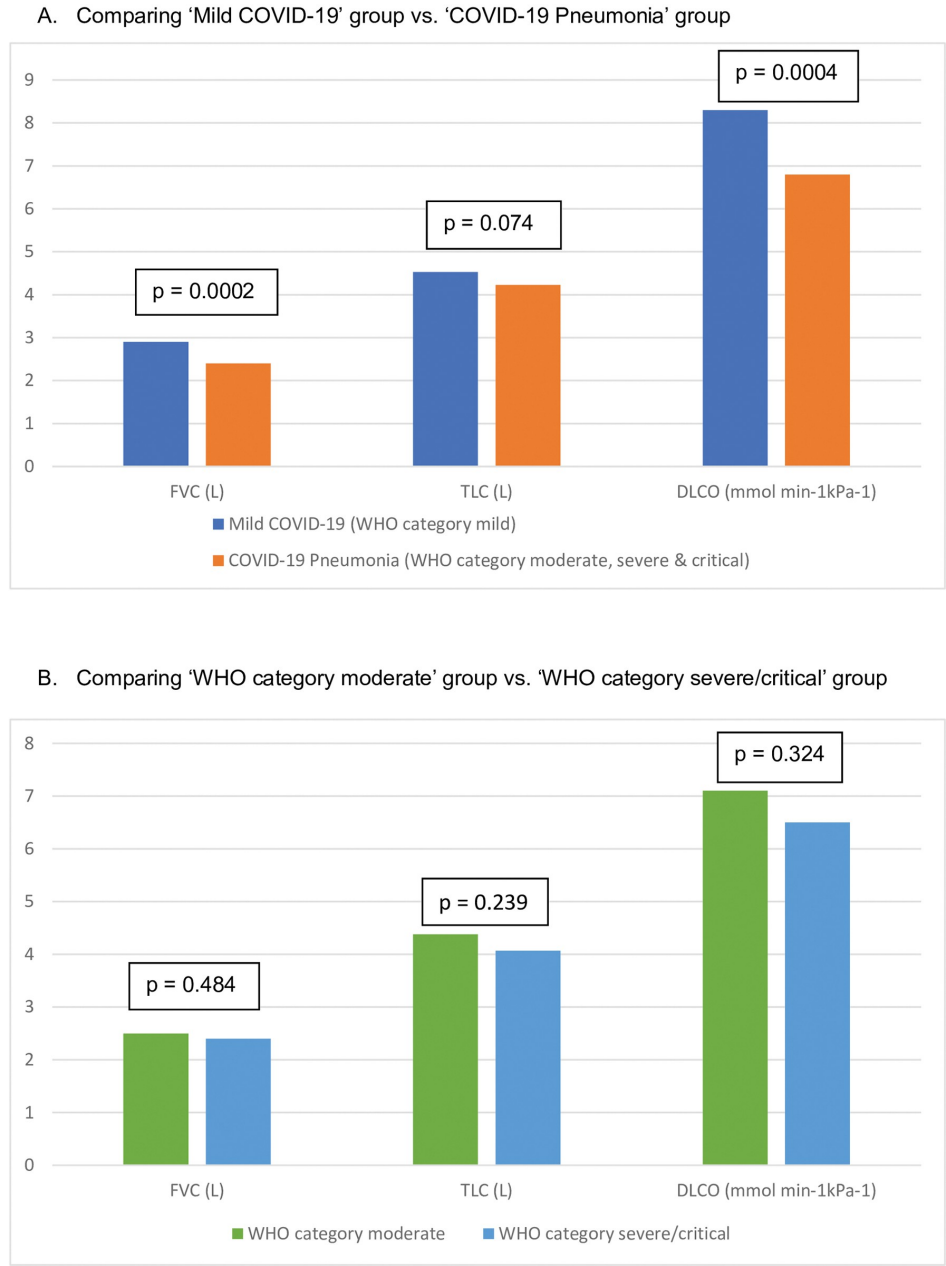
Comparison of the pulmonary function test outcomes across the disease severity.

The lung function parameters are compared across disease severity groups in Table 2 and Fig 1A & 1B. A total of 168 (81.2%) subjects were willing and able to perform spirometry testing, fulfilling technical requirements, while the measurement of TLC was successfully obtained in 160 (77.3%) and diffusing capacity (DLCO) measurement in 153 (73.9%) subjects.

**Table 2:** Comparison of pulmonary function test parameters and their abnormalities across disease severity groups.

Parameters	Total	‘Mild COVID-19’	‘COVID-19 Pneumonia’	P value	WHO Moderate	WHO Severe/ Critical	P value
**SPIROMETRY,** L (SD), N = 168							
FVC	2.71 (0.82)	2.9 (0.83)	2.4 (0.72)	0.0002	2.5 (0.63)	2.4 (0.78)	0.484
FVC (%)	83.93 (20.32)	88.18 (19.74)	77.85 (19.71)	0.001	82.61 (20.69)	73.61 (18.03)	0.056
FVC < 80%	60 (35.7)	23 (23.2)	37 (53.6)	<0.0001	10 (31.3)	27 (73.0)	0.001
FEV1	2.22 (0.73)	2.36 (0.76)	2.00 (0.62)	0.0014	2.00 (0.55)	2.01 (0.69)	0.925
FEV1 (%)	89.24 (23.47)	92.10 (23.46)	85.15 (23.04)	0.057	88.54 (26.11)	82.11 (19.78)	0.247
FEV1 < 80%	45 (26.7)	20 (20.2)	25 (36.2)	0.031	9 (28.1)	16 (43.2)	0.263
FEV1/FVC (%)	81.41 (7.93)	80.94 (8.19)	82.07 (7.55)	0.365	79.59 (7.08)	84.22 (7.36)	0.01
FEV1 / FVC < 70%	14 (8.3)	8 (8.1)	6 (8.7)	0.903	5 (15.6)	1 (2.7)	0.07
**LUNG VOLUMES,** L (SD), N = 160							
TLC	4.41 (1.05)	4.53 (1.03)	4.23 (1.04)	0.074	4.38 (1.02)	4.07 (1.06)	0.239
TLC (%)	84.45 (14.20)	87.91 (12.64)	79.48 (14.93)	0.0002	84.63 (11.63)	74.16 (16.24)	0.0045
TLC < 80%	56 (35.0)	24 (25.3)	32 (49.2)	0.004	10 (31.3)	22 (66.7)	0.008
RV/TLC	34.7 (7.5)	33.7 (7.5)	36.1 (7.6)	0.056	37.2 (6.8)	34.9 (7.7)	0.198
RV/ TLC (%)	118.1 (22.0)	117.1 (23.4)	119.4 (19.7)	0.525	120.6 (21.6)	118.2 (18.0)	0.632
RV/TLC ≥ 120%	69 (43.1)	37 (39.0)	32 (49.2)	0.197	19 (59.4)	13 (39.4)	0.107
**DIFFUSING LUNG CAPACITY,** mmol min^−1^ kPa^−1^ (SD) N = 153							
DLCO	7.7 (2.5)	8.3 (2.6)	6.8 (2.2)	0.0004	7.1 (2.2)	6.5 (2.1)	0.324
DLCO (%)	83.7 (20.9)	89.2 (18.7)	75.3 (21.4)	<0.0001	81.0 (20.7)	69.3 (20.7)	0.031
DLCO < 80%	68 (44.4)	29 (31.5)	39 (63.9)	<0.0001	17 (54.8)	22 (73.3)	0.133
**KCO**, mmol min^−1^ kPa^−1^ L^−1^ (SD) N = 153							
DLCO/Va	2.1 (0.4)	2.2 (0.3)	2.0 (0.4)	0.004	2.0 (0.4)	2.0 (0.3)	0.504
DLCO/Va (%)	109.3 (17.5)	111.8 (16.3)	105.6 (18.7)	0.032	108.8 (21.4)	102.3 (15.1)	0.18
DLCO/Va <80%	9 (5.9)	4 (4.4)	5 (8.2)	0.485	3 (9.7)	2 (6.7)	1.000

FEV1 (Forced Expiratory Volume at the end of the first second).

FVC (Forced Vital Capacity).

TLC (total lung capacity).

RV (Residual Volume).

DLCO (Diffusing capacity of lung for carbon monoxide).

DLCO/VA (Diffusing capacity adjusted for the alveolar volume ventilated).

In all, 35.7% subjects had low FVC (< 80%) and 35% had restrictive defect based on the criteria (TLC < 80%). While 8.3% had obstruction based on the criteria (FEV1/FVC <70%)—one previously diagnosed with COPD, seven with Asthma and one with Bronchiectasis. Five had no known pulmonary disease, never smoked or had other risk factors for COPD. Overall, 44.4% had impaired diffusing capacity. ‘COVID-19 pneumonia’ group had more subjects with reduction of FVC <80% (53.6% VS 23.2%; P<0.0001), reduction of FEV1 <80% (36.2% VS 20.2%; P = 0.031) and impairment of DLCO <80% (63.9% VS 31.5%; P<0.0001), compared to ‘mild COVID-19’ group. More subjects in the WHO category severe/critical group compared to the WHO category moderate group had FVC <80% (73% VS 31.3%; P = 0.001) and TLC <80% (66.7% VS 31.3%; P = 0.008).

The ‘COVID-19 pneumonia’ group, compared to ‘mild COVID- 19’ group had lower FVC (2.4 VS 2.9; P = 0.0002), FVC% (77.85 VS 88.18; P = 0.001), FEV1 (2.0 VS 2.36; P = 0.0014), TLC% (79.48 VS 87.91; P = 0.0002), DLCO (6.80 VS 8.30; P = 0.0004), DLCO% (75.30 VS 89.20; P<0.0001) and DLCO/VA (2.0 VS 2.2; P = 0.004). The WHO category severe/critical group had lower TLC% (74.16 VS 84.63; P = 0.0045) and DLCO % (69.30 VS 81.0; P = 0.031), compared to the WHO category moderate group.

### Six-minute walk test

**Fig 2:**
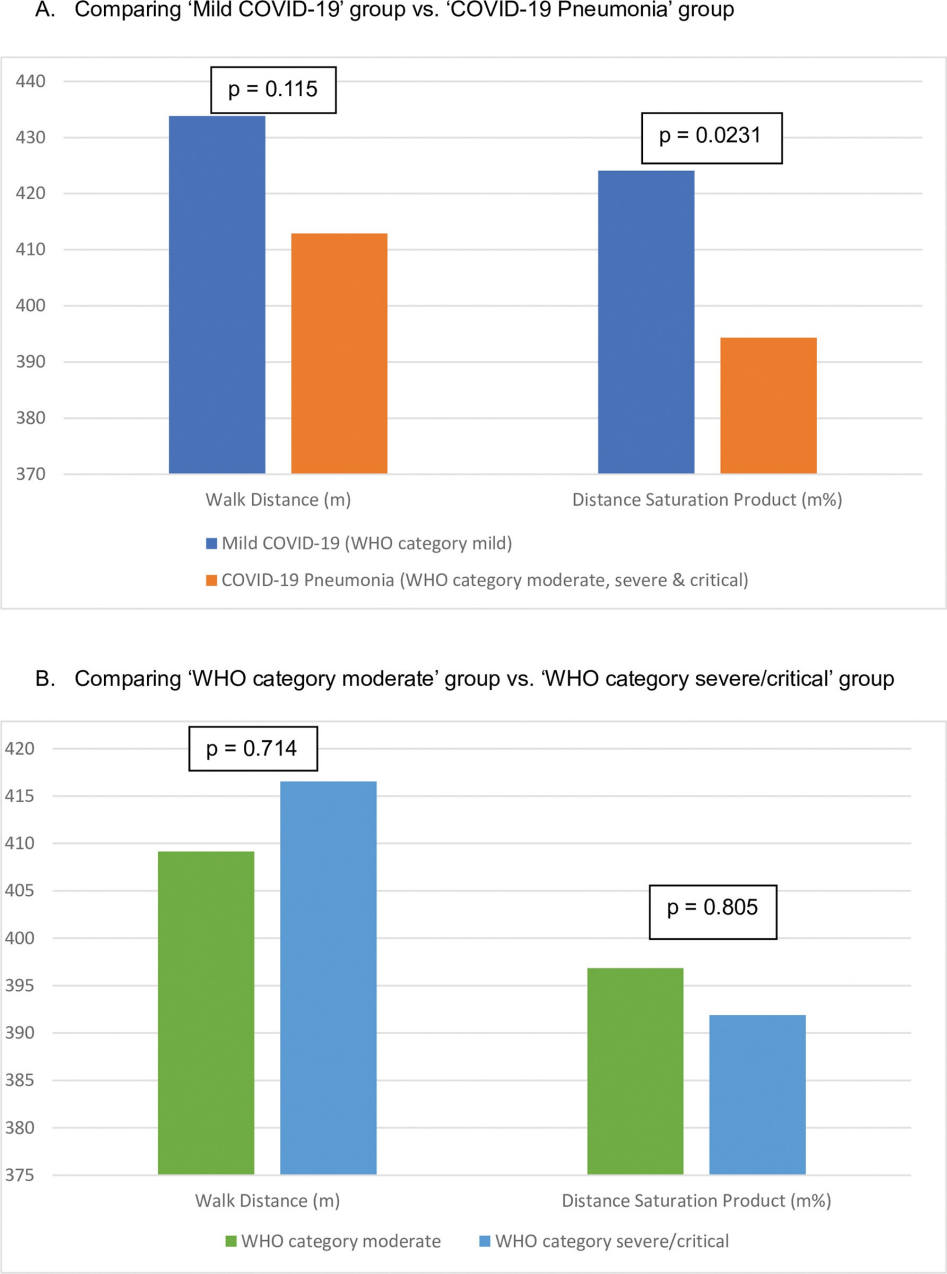
Comparison of the 6-minute walk test outcomes across the disease severity.

The results of the 6-minute walk test across disease severity groups are tabulated in Table 3 and Fig 2A & 2B. The subjects walked a mean distance of 425.26 meters, this represented (74.20%) of the predicted value. The mean lowest oxygen saturation was lower for the ‘COVID-19 pneumonia’ group compared to the ‘mild COVID-19’ group (94.89 VS 97.73; P<0.0001) and the drop in saturation ≥ 4% was higher (21.69% VS 4.84%; P = 0.001). However, there were no differences in the walk distance, the percentage of subjects who walked <80% of predicted and walk-work.

**Table 3:** Results of 6-minute walk test across disease severity groups.

Parameters, N = 198	Total	‘Mild COVID-19’	‘COVID-19 Pneumonia’	P value	WHO Moderate	WHO Severe/ Critical	P value
6MWD, m (SD)	425.26 (91.84)	433.83 (92.52)	412.89 (89.99)	0.115	409.15 (58.77)	416.54 (113.14)	0.714
6MWD (%), (SD)	74.20 (15.73)	72.91 (14.99)	76.07 (16.66)	0.166	78.20 (17.00)	73.99 (16.26)	0.258
6MWD (%) < 80%, [n (%)]	134 (67.68)	83 (70.94)	51 (62.96)	0.238	25 (62.50)	26 (63.41)	0.932
Distance saturation product, m% (SD)	411.92 (90.82)	424.09 (90.18)	394.35 (89.37)	0.0231	396.85 (57.28)	391.90 (112.97)	0.805
Walk work (WW), kgm (SD)	31905.57 (9960.66)	32220.19 (10209.29)	31451.12 (9634.82)	0.596	30725.03 (9132.16)	32519.51 (10613.99)	0.506
SpO2%, (SD)	96.57 (4.20)	97.73 (1.68)	94.89 (5.89)	<0.0001	96.48 (3.30)	93.34 (7.33)	0.016
SpO2 ≥ 4%, [n (%)]	24 (11.59)	6 (4.84)	18 (21.69)	0.001	2 (4.88)	16 (38.10)	<0.0001

6MWD (6-minute walk distance).

SpO2 (Oxygen saturation in blood).

### SGRQ scores

**Fig 3:**
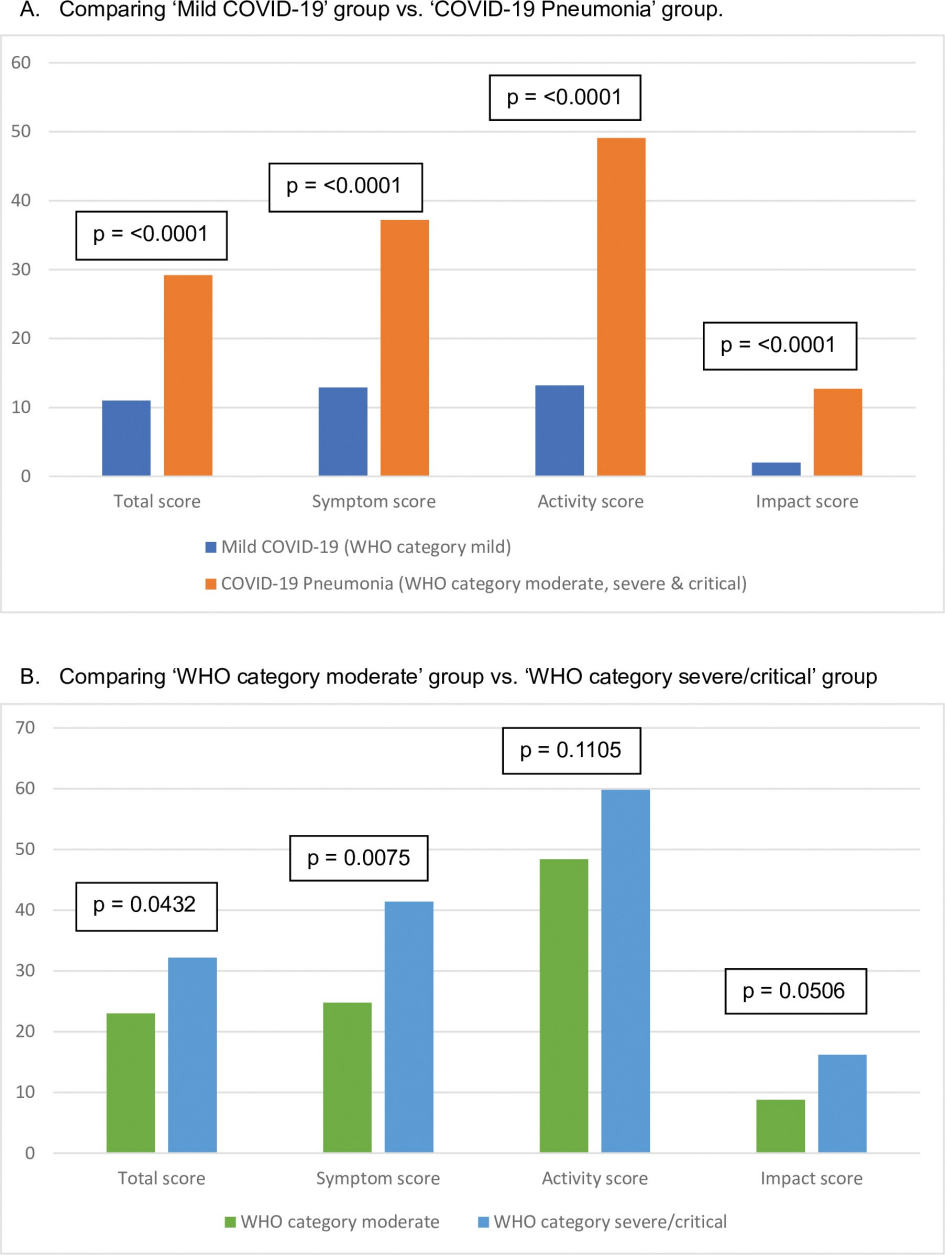
Comparison of the SGRQ outcomes across the disease severity.

The SGRQ scores are tabulated across disease severity groups in Table 4 and Fig 3A & 3B. The mean total score was higher for the ‘COVID-19 pneumonia’ group compared to the ‘mild COVID-19’ group (29.2 VS 11.0; P<0.0001). Likewise, all the domain scores were also higher for the ‘COVID-19 pneumonia’ group: symptom (37.2 VS 12.9; P<0.0001), activity (49.1 VS 13.2; P<0.0001) and impact (12.7 VS 2.0; P<0.0001). The WHO category severe/critical group had higher total scores than the WHO category moderate group (32.2 VS 23.0; P = 0.043) and also higher symptom scores (41.4 VS 24.8; P = 0.0075). Overall, more subjects in the ‘COVID-19 pneumonia’ group had severe symptoms (total scores ≥ 25) compared to the ‘mild COVID-19’ group (57.8% VS 29.5%; P<0.0001).

**Table 4:** SGRQ scores across disease severity groups.

Domains median(IQR) N = 205	Total	‘Mild COVID-19’	‘COVID-19 Pneumonia’	P value	WHO Moderate	WHO Severe/ Critical	P value
TOTAL	18.7 (2.0–36.1)	11.0 (1.1–30.2)	29.2 (13.7–43.3)	<0.0001	23.0 (6.4–41.3)	32.2 (21.2–49.2)	0.0432
Symptoms	21.9 (6.3–45.0)	12.9 (6.3–38.3)	37.2 (15.3–54.2)	<0.0001	24.8 (12.9–43.3)	41.4 (26.4–60.5)	0.0075
Activity	36.3 (0–61)	13.2 (0–53.5)	49.1 (18.6–72.8)	<0.0001	48.4 (0–66.8)	59.8 (35.7–72.8)	0.1105
Impact	7.3 (0–19.6)	2.0 (0–12.2)	12.7 (5.04–33.1)	<0.0001	8.8 (0–26.4)	16.2 (7.6–36.0)	0.0506
Total score ≥ 25 n (%)	84 (41.0)	36 (29.5)	48 (57.8)	<0.0001	18 (43.9)	30 (71.4)	0.011

### Chest radiology scores

The ‘mild COVID-19’ group is expected to have a RALE score of ‘zero’. Therefore, the comparison was made only between the WHO category moderate and the WHO category severe/critical groups. The median score was higher in the WHO category severe/critical compared to the WHO category moderate group (1.5 VS 0; P = 0.005).

## Discussion

To our knowledge, this was the first report looking at respiratory function in patients from southeast Asia, who have recovered from COVID-19 infection. Our cohort had a mean age of 48.7 years with a male preponderance (68.1%). In a cohort of Chinese subjects who were recruited for assessment of lung function, post-COVID-19, the mean age of the subjects was 49.1 years and 50% of them were females [7].

We looked at the key respiratory symptoms; dyspnoea was present in 49.3% and cough in 27.1%. A study by Sonnweber et al. in Austrian subjects revealed that 41% of all subjects exhibited persistent symptoms 100 days after COVID-19 onset—36% had dyspnoea and 17% cough [5]. Carfi et al. in an Italian cohort demonstrated that dyspnoea was present in 43% and cough in less than 20% of the subjects after a mean duration of around 60 days after the onset of the first COVID-19 symptom [12]. It appears that our subjects reported more symptoms than the European subjects. We also found that these symptoms were significantly higher in the ‘COVID-19 pneumonia’ group in comparison to the ‘mild COVID-19’ group, but there was no difference between the WHO category severe/critical and the WHO category moderate groups.

In a cohort of Chinese subjects studied by Mo et al., forty-four (40%) patients had at least one comorbidity, 23.6% patients had hypertension and 8.2% patients had diabetes [7]. Although of a similar age group, more of our subjects 72.5% had comorbidities and 40.1% had two or more comorbidities. The common comorbidities included diabetes mellitus (37.7%), systemic hypertension (33.3%) and chronic lung diseases (29.0%).

After the first study on Pulmonary function testing post-COVID-19 was reported by Mo et al. [7], the other studies with a substantive sample size were those by Huang et al. [13], Liu Kai et al. [14], Lv et al. [15], Lerum et al. [16], Daher et al. [17], Van den Borst et al. [18], and Sonnweber et al.[5] The first three were performed on Chinese subjects and the last four on European subjects. We are unaware of studies from Asia (other than China) and the rest of the world. To our knowledge, ours is one of the largest reports of the post-COVID-19 effects on lung function.

Thomas et al. combined the major studies which amounted to a total of 768 PFTs post COVID-19 [19]. The data indicated that DLCO is the main PFT parameter that was impaired after COVID-19 and is related to the severity of the disease; 152 patients (20.3%) with mild disease, 435 patients (32%) with moderate disease and 181 patients (59.5%) with severe disease had low DLCO. Conversely, spirometric parameters were well preserved, suggesting that a straightforward clinic-based pulmonary function test (e.g., using a hand-held spirometer) would not be able to identify or describe the residual effects of COVID-19 infection [19].

In our cohort, overall 44.4% had impairment of DLCO. The impairment was 31.5% in mild, 54.8% in moderate and 73.3% in severe groups. Using a definition of restriction as (TLC < 80%), 35.0% had restrictive defect and five patients who had obstructive ventilatory defect (FEV1/FVC <70%) had no known pulmonary disease and did not have smoking or any other risk factors for COPD. While this could be subclinical COPD, the role of COVID should also be kept in mind. Overall, it appeared that our subjects had more lung function impairment.

The difference in the impact of COVID-19 on different racial and ethnic groups is well described. Black, Hispanic, and AIAN people had experienced higher rates of COVID-19 infection and death compared to White people [20]. It appears that our cohort of Indian subjects had more comorbidities and had more impairment of lung function than the European and Chinese patients. In addition, studies have shown that disparities in Social Determinants of Health (SDOH) played a significant role in increased COVID-19 mortality, thus highlighting the social needs of low-income, low-education populations which impact equity in health outcomes [21].

Guler et al., in their cohort, with an average observation time of four months, demonstrated impairments in pulmonary function and physical performance. When compared to patients with mild and moderate illness, this was more pronounced in patients with prior severe and critical COVID-19 illness [22]. Specifically, TLC, FVC, FEV1, and DLCO were significantly lower in patients after severe/critical COVID-19, compared to patients after mild/moderate disease, and DLCO % predicted at four months was the most important, independent correlate of a more severe initial disease [22]. Our study has also demonstrated worse lung function in those with more severe disease.

Long-term lung function effects are not yet well studied. 145 patients participated in a prospective, multicenter, observational study assessing pulmonary function alterations at 60 days (visit one) and at 100 days (visit two) after the onset of COVID-19. They demonstrated that at 60 days, 42% had PFT impairment, of whom 27% had restrictive defects, TLC was reduced in 11% and DLCO was reduced in 31%. At 100 days, 36% of patients still showed impaired lung function; of whom 22% had restrictive abnormality, 11% had decreased TLC and 21% had decreased DLCO, suggesting that 10% of the patients who had decreased DLCO at 60 days had become normal by 100 days [5]. Zang et al. have shown that lung function recovery, which is characterized by an improvement from 6 months to 1 year after infection, and then a decline after the observed peak of recovery [23]. It is not yet known how many would be left with permanent PFT impairment due to residual lung fibrosis.

Tests evaluating exercise capacity have reported propensity for desaturation with exercise. Fuglebjerg et al. reported that the data of 26 patients at discharge, demonstrated that 50% of them had substantial desaturation (SpO2 < 90%) during the 6MWT on exertion [24]. We too demonstrated exercise desaturation, which correlated with severity. However, to our surprise, we did not find a reduction in the distance walked, distance saturation product and walk-work product.

Inflammation related acute lung injury could result in mortality of a significant proportion of patients with ARDS [25], while a large proportion of the survivors could experience permanent impairment of lung function and radiographic changes suggestive of pulmonary fibrosis [26,27]. Even a small degree of fibrosis in elderly patients with COVID-19, who may also have underlying lung disorders, could lead to considerable morbidity and mortality [28]. The true prevalence of post-COVID-19 pulmonary fibrosis would require long-term follow-up studies. However, given the large number of patients who have contracted the disease, it is likely that a large pool of patients would be left with fibrosis and persistent lung function impairment.

Carda et al. advocated pulmonary rehabilitation treatment based on the contention that it is usually recommended in lung fibrosis, and COVID-19 can also induce a restrictive lung disease [29]. Gloeckl et al. showed that pulmonary rehabilitation is a practically helpful way of improving exercise capacity, lung function and quality of life in those with COVID-19 related pulmonary function impairment [30]. There is no information yet on the benefit of pharmacotherapy agents, such as the use of steroids and anti-fibrotic drugs to prevent lung fibrosis and lung function impairment. Future studies should address these questions.

## Conclusion

In a large Indian cohort, we have reported the presence of post-COVID-19 residual lung damage, as assessed by lung function tests, exercise capacity, chest radiography and quality of life measurements. Our population reported relatively higher symptomatology and comorbidities and greater lung function impairment, compared to most published studies. We have shown that post-COVID-19 lung damage results in significant impairment of lung function, quality of life and effort tolerance.

### Limitations

Our study was conducted during the first wave of the pandemic in India and so the findings may not be extrapolatable to those affected by the subsequent waves. We evaluated our subjects after a mean duration of 63 days from the onset of their symptoms and were unable to follow up with our subjects for a longer period of time, till the damage stabilized.

### What is the new and future research direction?

We have confirmed the reports from other studies that COVID-19 causes impairment in lung function, quality of life and effort tolerance. Notably, our study shows that the Indian subjects developed worse impairment in lung function than other published cohorts mostly with Caucasian subjects. It would be interesting to know how many are left with permanent lung function damage and if this is also larger in the Indian population.

### Recommendations

Health systems should gear up to manage patients with post-COVID-19 lung damage.

## References

[1] Zhu N, Zhang D, Wang W, Li X, Yang B, Song J, et al. A Novel Coronavirus from Patients with Pneumonia in China, 2019. N Engl J Med 2020;382:727–33. doi: 10.1056/NEJMoa2001017 31978945 PMC7092803

[2] MoHFW | Home [Internet]. [cited 2023 June 1]. Available from: https://www.mohfw.gov.in/.

[3] Ooi GC, Khong PL, Müller NL, Yiu WC, Zhou LJ, Ho JC, et al. Severe Acute Respiratory Syndrome: Temporal Lung Changes at Thin-Section CT in 30 Patients. Radiology [Internet]. 2004 Mar [cited 2021 Feb 24];230(3). Available from: https://pubs.rsna.org/doi/abs/10.1148/radiol.2303030853. 10.1148/radiol.2303030853 14990845

[4] Zhang P, Li J, Liu H, Han N, Ju J, Kou Y, et al. Long-term bone and lung consequences associated with hospital-acquired severe acute respiratory syndrome: a 15-year follow-up from a prospective cohort study. Bone Res [Internet]. 2020 Feb [cited 2021 Feb 24];8:8. Available from: https://www.ncbi.nlm.nih.gov/pmc/articles/PMC7018717/. doi: 10.1038/s41413-020-0084-5 32128276 PMC7018717

[5] Sonnweber T, Sahanic S, Pizzini A, Luger A, Schwabl C, Sonnweber B, et al. Cardiopulmonary recovery after COVID-19: an observational prospective multicentre trial. Eur Respir J 2021;57:2003481. doi: 10.1183/13993003.03481-2020 33303539 PMC7736754

[6] Thille AW, Esteban A, Fernández-Segoviano P, Rodriguez J, Aramburu J, Vargas-Errazuriz P, et al. Chronology of histological lesions in acute respiratory distress syndrome with diffuse alveolar damage: a prospective cohort study of clinical autopsies. The Lancet Respiratory Medicine 2013;1:395–401. doi: 10.1016/S2213-2600(13)70053-5 24429204

[7] Mo X, Jian W, Su Z, Chen M, Peng H, Peng P, et al. Abnormal pulmonary function in COVID-19 patients at time of hospital discharge. Eur Respir J 2020;55:2001217. doi: 10.1183/13993003.01217-2020 32381497 PMC7236826

[8] COVID-19 Clinical management: living guidance [Internet]. [updated 2021 Jan 25; cited 2021 Jul 16]. Available from: https://www.who.int/publications-detail-redirect/WHO-2019-nCoV-clinical-2021-2.

[9] ATS standards for 6MWT.pdf [Internet]. [updated 2002 Mar; cited 2021 Mar 25]. Available from: https://www.atsjournals.org/doi/pdf/10.1164/ajrccm.166.1.at1102.

[10] Warren MA, Zhao Z, Koyama T Bastarache JA, Shaver CM, Semler MW, et al. Severity Scoring of Lung Edema on the Chest Radiograph Is Associated with Clinical Outcomes in ARDS. Thorax 2018;73:840–6. doi: 10.1136/thoraxjnl-2017-211280 29903755 PMC6410734

[11] 2023 GOLD Reports [Internet]. Global Initiative for Chronic Obstructive Lung Disease—GOLD. [updated 2023; cited 2023 June 5]. Available from: https://goldcopd.org/2023-gold-reports.

[12] Carfì A, Bernabei R, Landi F, for the Gemelli Against COVID-19 Post-Acute Care Study Group. Persistent Symptoms in Patients After Acute COVID-19. JAMA 2020;324:603. doi: 10.1001/jama.2020.12603 32644129 PMC7349096

[13] Huang Y, Tan C, Wu J, Chen M, Wang Z, Luo L, et al. Impact of coronavirus disease 2019 on pulmonary function in early convalescence phase. Respiratory Research 2020;21:163. doi: 10.1186/s12931-020-01429-6 32600344 PMC7323373

[14] Liu K, Zhang W, Yang Y, Zhang J, Li Y, Chen Y. Respiratory rehabilitation in elderly patients with COVID-19: A randomized controlled study. Complementary Therapies in Clinical Practice 2020;39:101166. doi: 10.1016/j.ctcp.2020.101166 32379637 PMC7118596

[15] Lv D, Chen X, Wang X, Mao L, Sun J, Wu G, et al. Pulmonary function of patients with 2019 novel coronavirus induced-pneumonia: a retrospective cohort study. Annals of Palliative Medicine 2020;9:3447452–3443452. doi: 10.21037/apm-20-1688 33065795

[16] Lerum TV, Aaløkken TM, Brønstad E, Aarli B, Ikdahl E, Lund KMA, et al. Dyspnoea, lung function and CT findings 3 months after hospital admission for COVID-19. Eur Respir J 2021;57:2003448. doi: 10.1183/13993003.03448–2020 33303540 PMC7736755

[17] Daher A, Balfanz P, Cornelissen C, Müller A, Bergs I, Marx N, et al. Follow up of patients with severe coronavirus disease 2019 (COVID-19): Pulmonary and extrapulmonary disease sequelae. Respiratory Medicine 2020;174:106197. doi: 10.1016/j.rmed.2020.106197 33120193 PMC7573668

[18] van den Borst B, Peters JB, Brink M, Schoon Y, Bleeker-Rovers CP, Schers H, et al. Comprehensive health assessment three months after recovery from acute COVID-19. Clin Infect Dis 2020;ciaa1750. 10.1093/cid/ciaa1750. PMC7717214 33220049

[19] Thomas M, Price OJ, Hull JH. Pulmonary function and COVID-19. Curr Opin Physiol 2021;21:29–35. doi: 10.1016/j.cophys.2021.03.005 33817453 PMC7997144

[20] Hill L, Artiga S. Covid-19 cases and deaths by Race/ethnicity: Current data and changes over time [internet]. Coronavirus (COVID-19); 2022 Aug [cited on 2023,10 October]. Available from: https://www.kff.org/racial-equity-and-health-policy/issue-brief/covid-19-cases-and-deaths-by-race-ethnicity-current-data-and-changes-over-time.

[21] Pattath P. Social Determinants of Health and Racial/Ethnic Disparities in COVID-19 Mortality at the County Level in the Commonwealth of Virginia. Fam Community Health. 2023 Apr-Jun 01;46(2):143–150. doi: 10.1097/FCH.0000000000000330 36455199

[22] Guler SA, Ebner L, Aubry-Beigelman C, Bridevaux PO, Brutsche M, Clarenbach C, et al. Pulmonary function and radiological features 4 months after COVID-19: first results from the national prospective observational Swiss COVID-19 lung study. Eur Respir J 2021;57:2003690. doi: 10.1183/13993003.03690-2020 33419891 PMC8082329

[23] Zhang H, Li X, Huang L, Gu X, Wang Y, Liu M, et al. Lung-function trajectories in COVID-19 survivors after discharge: A two-year longitudinal cohort study. EClinicalMedicine. 2022 Sep 28;54:101668. doi: 10.1016/j.eclinm.2022.101668 36188433 PMC9514976

[24] Fuglebjerg NJU, Jensen TO, Hoyer N, Ryrsø CK, Lindegaard B, Harboe ZB. Silent hypoxia in patients with SARS CoV-2 infection before hospital discharge. International Journal of Infectious Diseases 2020;99:100–1. doi: 10.1016/j.ijid.2020.07.014 32663601 PMC7836996

[25] Meduri GU, Headley S, Kohler G, Stentz F, Tolley E, Umberger R, et al. Persistent Elevation of Inflammatory Cytokines Predicts a Poor Outcome in ARDS: Plasma IL-1β and IL-6 Levels Are Consistent and Efficient Predictors of Outcome Over Time. CHEST 1995;107:1062–73. 10.1378/chest.107.4.1062. 7705118

[26] Masclans JR, Roca O, Muñoz X, Pallisa E, Torres F, Rello J, et al. Quality of life, pulmonary function, and tomographic scan abnormalities after ARDS. Chest 2011;139:1340–6. doi: 10.1378/chest.10-2438 21330382

[27] Desai SR, Wells AU, Rubens MB, Evans TW, Hansell DM. Acute Respiratory Distress Syndrome: CT Abnormalities at Long-term Follow-up. Radiology 1999;210:29–35. doi: 10.1148/radiology.210.1.r99ja2629 9885583

[28] Spagnolo P, Balestro E, Aliberti S, Cocconcelli E, Biondini D, Casa GD, et al. Pulmonary fibrosis secondary to COVID-19: a call to arms? Lancet Respir Med 2020;8:750–2. doi: 10.1016/S2213-2600(20)30222-8 32422177 PMC7228737

[29] Carda S, Invernizzi M, Bavikatte G, Bensmaïl D, Bianchi F, Deltombe T, et al. The role of physical and rehabilitation medicine in the COVID-19 pandemic: The clinician’s view. Annals of Physical and Rehabilitation Medicine 2020;63:554–6. doi: 10.1016/j.rehab.2020.04.001 32315802 PMC7166018

[30] Gloeckl R, Leitl D, Jarosch I, Schneeberger T, Nell C, Stenzel N, et al. Benefits of pulmonary rehabilitation in COVID-19: a prospective observational cohort study. ERJ Open Res 2021;7:00108–2021. doi: 10.1183/23120541.00108-2021 34095290 PMC7957293

